# Aggressive sperm immobilization improves reproductive outcomes in patients with suboptimal semen parameters and previous ICSI fertilization failure

**DOI:** 10.1038/s41598-024-56092-4

**Published:** 2024-03-04

**Authors:** Ching-Wen Chou, Shee-Uan Chen, Chin-Hao Chang, Yi-Yi Tsai, Chu-Chun Huang

**Affiliations:** 1 Department of Obstetrics and Gynecology, National Taiwan University Hospital, Taipei, 100 Taiwan; 2 Department of Medical Research, National Taiwan University Hospital and National Taiwan University College of Medicine, Taipei, Taiwan

**Keywords:** Biological techniques, Medical research

## Abstract

Intracytoplasmic sperm injection (ICSI) is the most effective procedure to resolve male infertility, enhancing overall fertilization and pregnancy outcomes. However, it is important to note that fertilization failure (FF) can still occur in a few cases after ICSI. This study aims to introduce a specialized technique of aggressive sperm immobilization for ICSI and evaluate its impact on reproductive outcomes in cases involving prior fertilization failure. All infertile couples with male partners having suboptimal semen samples and previous ICSI fertilization failure were evaluated using retrospective data from National Taiwan tertiary university hospital (NTUH) between January 2016 and February 2022. Fertilization failure in our study was defined as less than 30% fertilization rate (FR, the number of normally fertilized oocytes divided by the total number of injected mature oocytes). Data involving both standard (routine procedure) and aggressive sperm immobilization (SI) techniques during different ICSI cycles were included in this study. Standard and aggressive SI methods were performed by compressing the distal half tail of the spermatozoa ≦ 5 and 15 times prior to ICSI respectively. Generalized estimating equations analysis were applied to compare the clinical outcomes between two procedures. Overall, data from 23 infertile couples who had undergone 65 ICSI cycles (31 standard SI with low fertilization rate and 34 aggressive SI) were included in the study. The average FR in the ICSI cycles with standard SI and aggressive SI were 23.6 ± 23.1% and 49.5 ± 31.8 respectively (*P* = 0.0002). The majority of embryos were transferred at the day 3 stage, with an average number transferred of 2.6 ± 0.9 in the aggressive SI group and 1.9 ± 0.9 in the standard group. The number of embryos transferred per transfer cycle was higher in the aggressive SI (*P* = 0.015), whereas the number of good-quality embryos was similar between the two procedures (*P* = 0.44). There were one and seven live births from the standard SI cycles and aggressive SI cycles respectively. In conclusion, aggressive SI was associated with a significantly higher FR, resulting in more available embryos for transfer without compromising embryo quality. Therefore, this specialized technique improved pregnancy outcome among infertile couples with a previous ICSI–FF. It can be a safe, economic, and effective method to improve the assisted reproductive technologies outcomes for infertile patients affected by previous ICSI–FF.

## Introduction

Intracytoplasmic sperm injection (ICSI) is the most effective and efficient assisted reproductive procedure to resolve male factor infertility. The overall fertilization rate (FR) after the ICSI procedure can be up to 80%. However, fertilization failure (FF) after ICSI has been reported in 1–5% of cases even with morphologically normal gametes^1–3^. Fertilization rates ranging from 0 to 30% have been consistently observed in repeated ICSI cycles for certain patients, in which was viewed as fertilization failure^4,5^. Several mechanisms associated with FF have been proposed and verified, including oocyte activation deficiency (OAD), premature sperm chromatin condensation, sperm aster defects, and oocyte spindle defects^6^. At the molecular level, sperm-induced intracytoplasmic calcium (Ca^2+^) oscillations are essential for oocyte activation. Therefore, one of the most commonly used treatment options for patients with FF after ICSI is assisted oocyte activation (AOA) using Ca^2+^ ionophores or strontium chloride (SrCl_2_)^7,8^. Several meta-analyses have compared the reproductive outcomes of ICSI following AOA with those of conventional ICSI^3,7,9–11^.

Significantly improved reproductive outcomes have been shown in the AOA group in two recent meta-analyses^7,10^, which revealed that AOA with Ca^2+^ ionophores after ICSI improved fertilization, cleavage, and implantation rates. However, these meta-analyses included different types of studies, including case reports, retrospective cohorts, prospective cohorts, and randomized controlled studies (RCTs). The study population was also heterogeneous, and severe male factor infertility patients without a history of ICSI FF were included in these analyses^7,10^. Another meta-analysis that included only RCTs showed that AOA might be associated with an increase in the number of cleavage-stage and high-quality embryos^9^. Nevertheless, there is insufficient evidence to support the efficacy and safety of AOA before ICSI, due to the different AOA methods and heterogeneous patient inclusion criteria used in the studies^9^. In addition, concerns regarding the possible mutagenic and epigenetic effects of AOA on oocytes and embryos have been raised, and further investigation is required^11,12^.

Plasma membrane damage in sperms has been shown to be an essential process in sperm immobilization (SI) before ICSI and plays a vital role in sperm-triggered oocyte activation^13,14^. Damage to the sperm membrane can induce gradual disruption of other parts of the sperm membrane and allow entry and activation of sperm nucleus-decondensing factor which is released from the oocyte. Subsequently, swelling of the sperm head can cause membrane rupture, and sperm-associated oocyte-activating factors can be released into the ooplasm to induce oocyte activation^15–17^. Therefore, SI prior to ICSI is thought to be routine protocol and to be necessary for efficient fertilization^18^.

Sperm immobilization (SI) with pipetting method is often employed in ICSI and improves fertilization and pregnancy rates^13,15,18^. However, no previous studies have investigated the effect of the aggressive SI method on assisted reproductive technologies (ART) outcomes in couples with ICSI FF. Our hypothesis was that aggressive SI prior to ICSI might enhance sperm plasma membrane damage and promote oocyte activation, especially among infertile couples with suboptimal semen samples and a history of ICSI FF. This was a retrospective cohort analysis of infertile couples who experienced FF during previous ICSI cycles and then received subsequent ICSI cycles with aggressive SI. As some patients underwent more than one ICSI cycle in our study, generalized estimating equations (GEE) for repeated measurement analysis were applied to adequately compare the clinical outcomes between standard and aggressive SI prior to ICSI.

## Methods

### Study population and ethical approval

This retrospective, observational, single-center cohort study was conducted at a tertiary university hospital. Patients between January 2016 and February 2022 received ART with standard SI prior to ICSI due to male factor infertility, as defined by the World Health Organization 2010 (WHO 5th Edition) criteria^19,20^. In cases where couples are experiencing infertility and the male partner exhibits suboptimal semen samples, male factor is often identified as a possible contributing factor of infertility. Oligozoospermia refers to male sperm concentration below the lower reference limit of 15 million sperm/mL. It was also further classified as severe (less than 5 million sperm/mL), moderate (5–10 million sperm/mL), or mild (10–15 million sperm/mL) according to Shaw’s Textbook of Gynaecology (16th Edition)^21^. Asthenozoospermia was defined as < 40% total motility or < 32% progressive motility.

FR was defined as the number of normally fertilized oocytes with two pronuclei divided by the total number of injected mature oocytes). In our study, a fertilization rate of below 30% was selected as an indicator of fertilization failure, following the definition of fertilization failure from previous studies^4,5^. All of the enrolled patients experienced ICSI FF of any ICSI cycles. Because of FF of any ICSI cycles, aggressive SI was then applied in subsequent ICSI cycles in order to improve fertilization in these patients.

**Figure 1:**
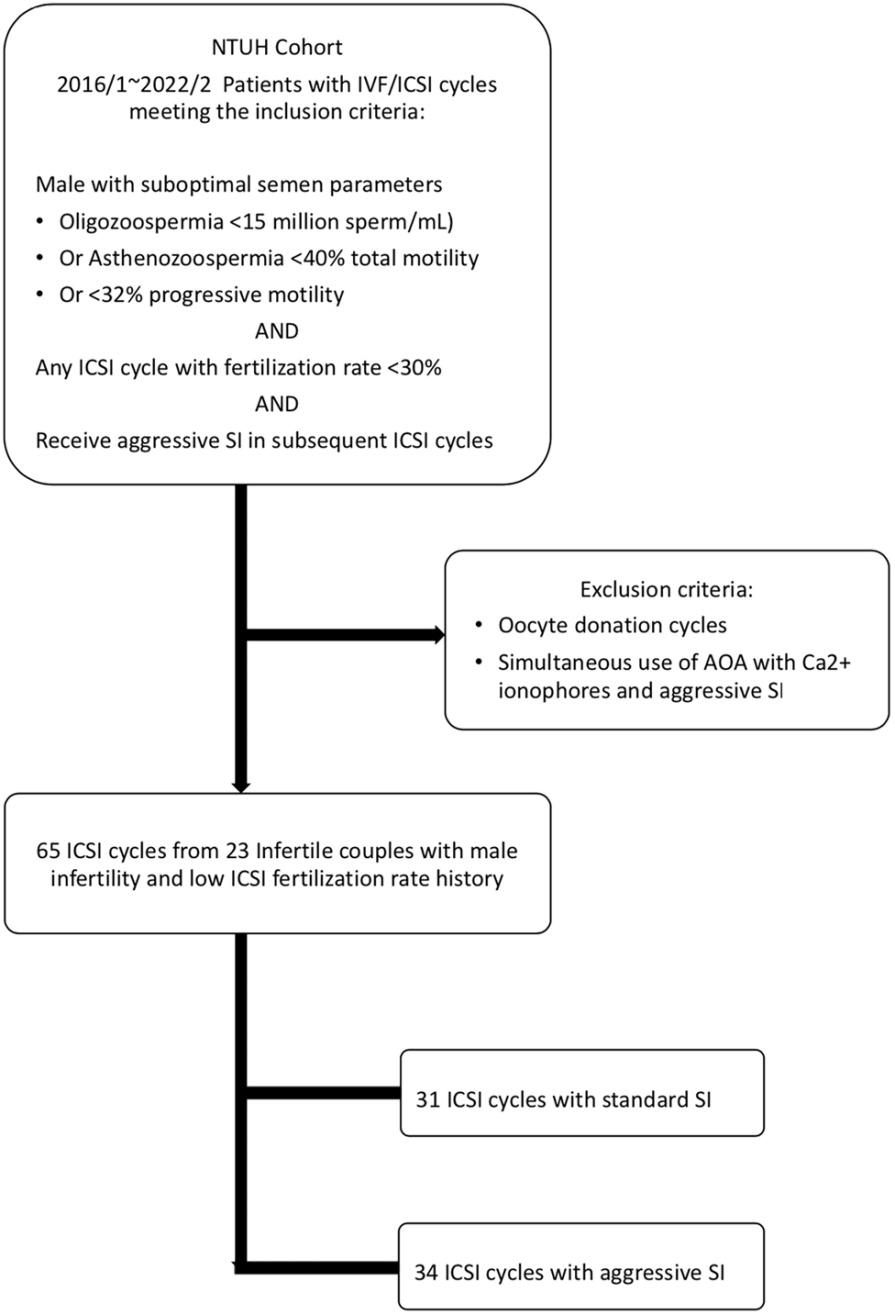
Study flow chart of patient enrollment from NTUH cohort retrospectively. *NTUH* National Taiwan University Hospital, *IVF* in vitro fertilization, *ICSI* intracytoplasmic sperm injection, *AOA* assisted oocyte activation, *SI* sperm immobilization.

The patients who underwent oocyte donation cycles were excluded from the study. Couples with the simultaneous use of AOA with Ca^2+^ ionophores and aggressive SI were also excluded (Fig. 1). This study was approved by the Ethics Committee of National Taiwan University Hospital (IRB No. 202212023RIND) and was also approved a request to waive the documentation of informed consent by the Ethics Committee of National Taiwan University Hospital. All patient data was performed by de-identification analysis. All methods were carried out in accordance with relevant guidelines and regulations.

### Ovarian stimulation

Pituitary downregulation was performed using either a gonadotropin-releasing hormone (GnRH) agonist protocol (Lupro®, leuprolide acetate, Nang-Kuang Co., Tainan, Taiwan) or a GnRH antagonist protocol (Orgalutran®, Ganirelix, Organon, Dublin, Ireland), as described in our previous study^22^. Ovarian stimulation was achieved using recombinant FSH (rFSH; Gonal-F®; Merck-Serono, Geneva, Switzerland) or highly purified human menopausal gonadotropin (hp-hMG; Menopur®, Ferring Pharmaceuticals, Geneva, Switzerland) containing 75 IU/ampule of FSH and LH activity. The selection of the protocol and type of gonadotropin used were individualized according to the characteristics of each patient and the clinician’s preference. The starting dose of gonadotropins ranged from 200 to 300 IU and was individualized according to the patient’s body weight (> 60 kg, 200 IU; > 75 kg, 250 IU; > 90 kg, 300 IU). When the mean diameter of the leading follicles reached 18 mm and at least two follicles had corresponding serum estradiol concentrations (each follicle contributing to estradiol 150–200 pg/mL), the follicles were triggered. Notably, 34–36 h after human chorionic gonadotropin administration, oocytes were retrieved via transvaginal ultrasound-guided aspiration.

### Preparation of sperm and oocytes

Motile spermatozoa were obtained using two-layer (90% and 45% gradients; VitroLife) discontinuous gradient centrifugation. Each sample was washed and resuspended in human tubal fluid (HTF) medium supplemented with 10% serum protein substitute (SPS and SAGE) and then kept in an incubator at 37 °C with 5% CO_2_ in the air for swim-up for a duration of 1 h^23^. For the oocytes, their cumuli were removed and nude oocytes were transferred into HTF containing 80 IU hyaluronidase (Sigma H3884). The oocytes were then washed in HTF medium and transferred back to SAGE fertilization medium. Metaphase II (MII) oocytes with their first polar bodies were collected for microinjection.

### Sperm immobilization and ICSI procedures

**Figure 2:**
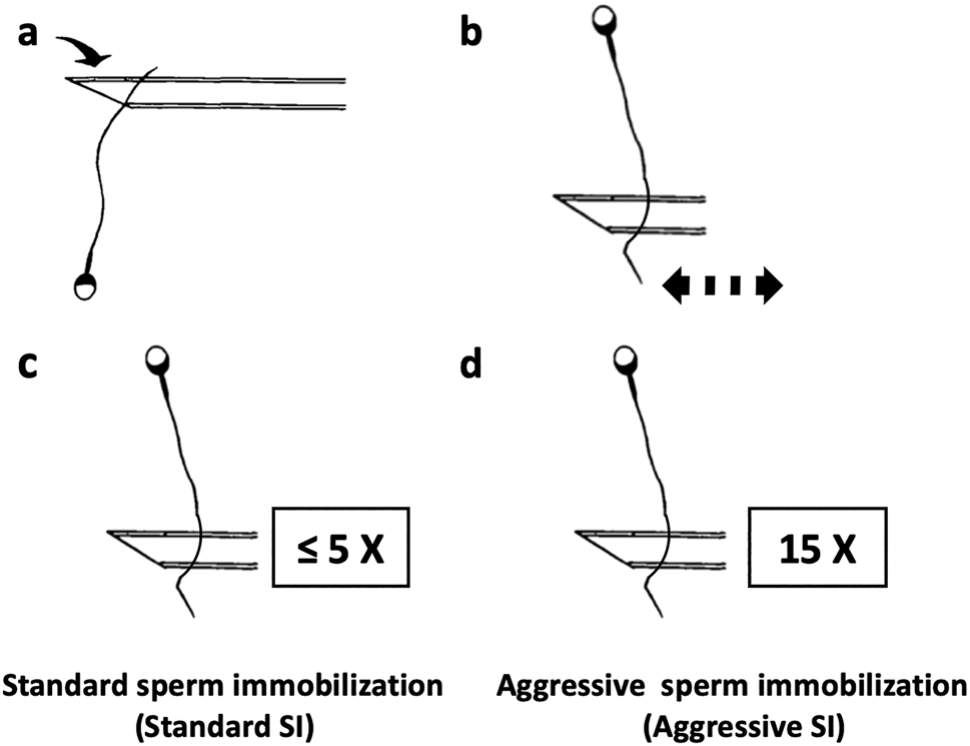
Compressing the tail at the tip (**a**) the tip of the tail is pressed with the injection micropipette by operating the three-dimensional joystick. (**b**) The micropipette is quickly operated in the perpendicular direction to the distal half of tail and away from the mid-piece region with forward/backward pressing movement. (**c**) Standard procedure: pressing the distal half of tail against the lid of the dish with a microinjection pipette less than or equal to five times until sperm immobilization. (**d**) More aggressive procedure: based on application of the standard procedure but compressing at the tip up to 15 times.

The micromanipulation procedure was conducted on the lid of a petri dish (Falcon 3001) using HEPES-buffered culture medium with 0.5% human serum albumin (Sigma A1653), incubated at 37 °C with 5% CO_2_ in the air. Prior to ICSI, one drop of motile spermatozoa (± 1–2 μL) was placed in the 10% polyvinylpyrrolidone (PVP). Micromanipulation was performed using a microscope (Nikon) and spermatozoa with grossly normal morphology were selected for the injection micropipette. The injection micropipette was used to press against distal half of tail via the three-dimensional joystick and subsequently was performed in the perpendicular direction to the tail forward and backward. When the tip of the sperm tail was pressed, the spermatozoa became permanently immobilized^24^. Routine SI was performed by above mentioned procedure and the sperm pressing times varied from one to five times (less than or equal to five times) as a means of standard sperm immobilization before ICSI in our laboratory. Although SI has been part of routine procedure before ICSI^18^, the degree of SI is not been standardized. In this study, aggressive SI was performed by compressing the tail of the spermatozoa at the tip up to 15 times (Fig. 2). Subsequently, the first polar body of the MII oocyte was placed in the 12 or 6-o’clock position and the immobilized sperm was injected into the oocyte by advancing the injection micropipette to the 3-o'clock position. ICSI was performed following 39–41 h after oocyte triggering^25,26^.

### Embryo transfer

A good-quality embryo on day 3 was defined as at least 6-cell stage embryos with less than 10% fragmentation and equally or slightly unequally sized blastomeres^27^. The blastocyst stage was evaluated on day 5 based on the tightly packed inner cell mass, good trophectoderm layer, and degree of expansion^28,29^. The same criteria were applied for both standard and aggressive SI techniques. In this study, we followed the principles of American Society for Reproductive Medicine^30^ guidelines about recommended limits of the embryos transferred numbers: age < 35, number ≤ 1; age 35–37, strong consideration for 1 embryo; age 38–40, number ≤ 3 cleavage-stage embryos or 2 blastocysts; and age 41–42, number ≤ 4 cleavage stage embryos or 3 blastocysts^30^.

### Outcome measures

The evaluated patient characteristics included female age, male age, body mass index (BMI), parity at initial enrollment, cause of infertility, baseline hormone levels, semen analysis data, ART protocols, time from oocyte triggering to ICSI, and the number of embryos transferred. The primary outcome was the LBR. The secondary outcomes included the clinical pregnancy rate, defined as the presence of fetal heartbeat at the 7th week gestational age, FR (the number of normally fertilized oocytes with two pronuclei divided by the total number of injected mature oocytes), and the number of transferred and good-quality embryos per transfer cycle.

### Statistical analysis

All outcomes were explored for potential associations with age, sperm tail pressing method, time interval between oocyte triggering and ICSI, gravidity, and the number of retrieved MII oocytes using GEE. The GEE model was applied to estimate the parameters of a generalized linear model with possible correlations between the subject outcomes at different time points in the same or different patients^31,32^. All statistical analyses were performed using the SAS software (version 9.4)^33^. Significant variables (*P* < 0.05) in the univariate model were included in the multivariate model. The significance level was set at *P* < 0.05.

## Results

### Characteristics of study population and ART treatments

A total of 23 couples with suboptimal semen samples and experiencing ICSI-FF were recruited. Their baseline characteristics are shown in Table 1. The average female age and male age was 38.9 ± 5.9 and 42.8 ± 6.2 years, respectively, at the start of the ART treatments, and the indication of ICSI procedure was male factor infertility in all cases. Twelve (52.2%) patients also had ovarian factor infertility. Overall, 52.1% of couples presented with oligozoospermia, and 65.2% of couples presented with asthenozoospermia (Table 1).

**Table 1:** Baseline characteristics of twenty-three infertile couples.

Patient characteristics	Number of patients (n = 23)
Female age (years)^a^	38.9 ± 5.9
Male age (years)^a^	42.8 ± 6.2
Female BMI^a^	25.2 ± 4.2
Parity at the initial enrollment	
Nulliparous	23
Parous	0
Infertility cause^b^	
Male factors	23 (100%)
Tubal factors	0 (0%)
Ovarian factors	12 (52.2%)
Endometriosis	1 (4.3%)
Baseline hormone level^a^	
FSH (mIU/mL)	8.5 ± 3.6
E2 (pg/mL)	40.9 ± 18.3
Semen analysis^b^	
Oligozoospermia	
Mild	3 (13%)
Moderate	5 (21.7%)
Severe	4 (17.4%)
Asthenozoospermia	15 (65.2%)

*BMI* body mass index*, FSH* Follicle-stimulating hormone*, E2* Estradiol.

Mild oligozoospermia (10–15 million sperm/mL), moderate oligozoospermia (5–10 million sperm/mL), severe oligozoospermia (< 5 million sperm/mL)^21^.

Asthenozoospermia (< 40% total sperm motility or < 32% progressive motility)^19,20^.

^a^The values were presented as mean and standard deviation.

^b^The data were expressed as total patient number and percentage.

The characteristics and treatment outcomes of ART-ICSI cycles grouped according to aggressive and standard SI methods are shown in Table 2. Similar results were observed between the two groups for female age, male age, sperm concentration, total sperm motility, time from oocyte triggering to ICSI, and number of good-quality embryos transferred per transfer cycle. There was also no significant difference in the stimulation protocols, duration of stimulation and dose of recombinant FSH between the standard and the aggressive SI group. GnRH antagonist protocols were utilized in most ART cycles and similar distribution between the standard and the aggressive SI groups. Only few patients underwent GnRH agonist protocols.

**Table 2:** Characteristics and outcomes of ART cycles grouped by aggressive and standard sperm immobilization groups in twenty-three infertile couples.

	Aggressive sperm immobilization	Standard sperm immobilization
Number of ICSI cycles	34	31
GnRH agonist protocol	5	6
GnRH antagonist protocol	29	25
Female age (years)^a^	39.44 ± 5.03	38.35 ± 6.77
Male age (years)^a^	43.29 ± 5.97	42.16 ± 6.52
Semen parameter (pre-wash)		
Concentration (× 10^6^/mL)^b^	20 (8.3–50.3)	19 (1.5–43)
Total motility (%)^a^	29.9 ± 20.7	25.7 ± 23.3
Number of MII oocytes retrieved per ICSI cycle^a^	9.21 ± 8.02	6.23 ± 4.01
Total 2PN embryos	133	47
Number of 2PN embryos per ICSI cycle^a^	3.9 ± 3.1	1.5 ± 1.4
Fertilization rate (%)^a^	49.5 ± 31.8	23.6 ± 23.1
Total ET cycles (n)	23	18
Day 3 ET cycles	21	17
Day 5 ET cycles	2	1
Number of embryos transfer per ET cycle^a^	2.6 ± 0.9	1.9 ± 0.9
Number of good-quality embryos transferred per ET cycle^a^	1.1 ± 1.2	0.9 ± 0.8
Clinical outcome per ICSI cycle^c^		
Clinical pregnancy	7 (20.6%)	1 (3%)
Live birth	7 (20.6%)	1 (3%)
Clinical outcome per ET cycle^c^		
Clinical pregnancy	7 (30.4%)	1 (5.6%)
Live birth	7 (30.4%)	1 (5.6%)

*GnRH* gonadotropin-releasing hormone*, ICSI* intracytoplasmic sperm injection*, HCG* human chorionic gonadotropin, *MII* metaphase II, *ET* embryo transfer.

^a^The values were presented as mean and standard deviation.

^b^The data were expressed as median and interquartile range.

^c^The data were expressed as total number and percentage.

A total of 65 ART-ICSI cycles were performed (31 traditional ICSI cycles with standard SI and 34 ICSI cycles with aggressive SI). In this study, the majority of embryos were transferred at the day 3 stage. There were twenty-one day 3 embryo transfer (ET) cycles in the aggressive SI group and seventeen day 3 ET cycles in the standard SI group. Only three cases involved the utilization of day 5–6 embryos, with two in the aggressive SI group and one in the standard SI group. Due to the predominant practice of transferring embryos at the early day 3 stage, limited data were available regarding day 5 blastocyst transfers in our study. Aggressive SI can significantly improve FR, which lead to the more available number of embryos transferred per ET cycle, and eventually, the higher LBR in patients with a history of ICSI FF.

### The reproductive outcomes in the aggressive sperm immobilization procedure

The reproductive outcomes are shown in Table 2. In the standard SI group, 193 MII oocytes were retrieved, resulting in 47 fertilized 2PN embryos (overall FR, 24.4%). The average FR in the standard SI group was 23.6 ± 23.1% per retrieval cycle. A total of 18 transfer cycles were performed, and the average number of transferred embryos was 1.9 ± 0.9. Only one patient had a clinical pregnancy, resulting in a live birth. In the aggressive SI group, 313 MII oocytes were retrieved, resulting in 133 fertilized 2PN embryos (overall FR, 42.5%). The average FR in the aggressive SI group was 49.5 ± 31.8% per retrieval cycle. Overall, 23 transfer cycles were performed. Seven patients eventually became pregnant, with seven live births. More embryos were available for transfer in the aggressive sperm immobilization group, and the average number of transferred embryos was 2.6 ± 0.9.

**Figure 3:**
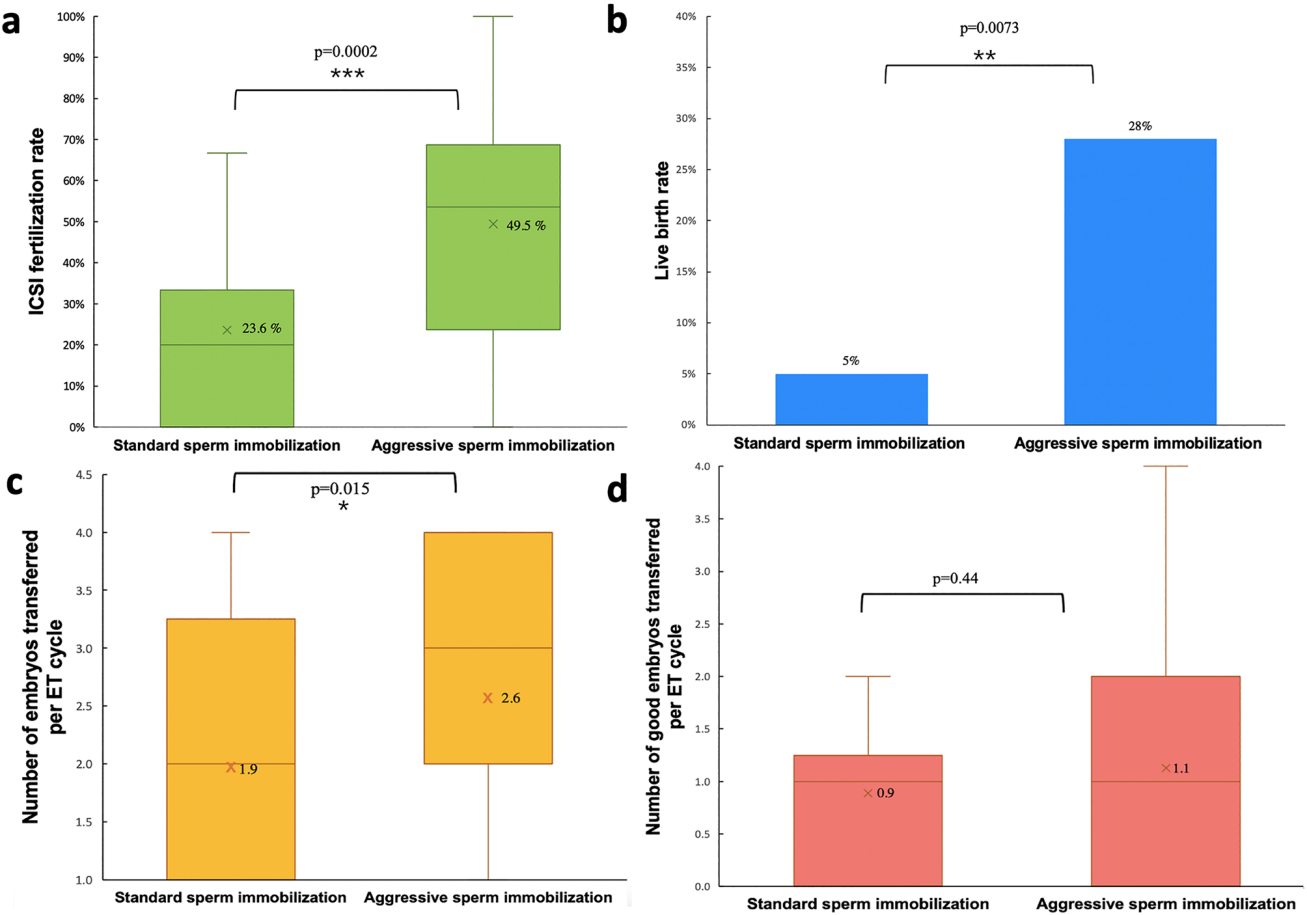
Box plot illustration of the first to third quartiles of ART outcomes. The transverse line travels through the box at the median. The cross-marks represent the mean. (**a**) ICSI fertilization rate, (**b**) live birth rate, (**c**) number of embryos transferred per ET cycle, and (**d**) number of good-quality embryos per ET cycle, grouped by the sperm immobilization method. The *P* value was calculated via a GEE multi-regression analysis and adjusted for age, sperm immobilization method, time interval between oocyte triggering and ICSI, gravidity, parity, and number of retrieved MII oocytes.

In a univariate regression analysis using the GEE method (Table 3), both aggressive SI and female age were associated with a significantly higher ICSI FR. There was no significant correlation between the ICSI FR and other parameters, including gravida, number of retrieved oocytes, or duration from oocyte trigger to ICSI. The significance of age diminished after adjusting for the multivariate analysis (*P* = 0.1297). However, aggressive SI prior to ICSI was associated with an increase of 27.4% in the FR compared to traditional ICSI with standard SI (estimated 95% CI 13.1–41.8%, *P* = 0.0002) (Table 3, Fig. 3a), after adjusting the factors of female age. In terms of live births, the application of aggressive SI, female age, and the number of retrieved oocytes showed a significant correlation in the univariate regression analysis. After adjustment with multivariate analysis, only the application of aggressive SI was associated with a significantly higher LBR (*P* = 0.0073; OR 23.45 with 95% CI 23.39–23.52 by GEE multi-regression analysis) (Table 3, Fig. 3b). In addition, the number of embryos transferred per transfer cycle was higher in the aggressive SI (*P* = 0.015, Fig. 3c), whereas the number of good-quality embryos was similar between the two procedures (*P* = 0.44, Fig. 3d).

**Table 3:** Generalized estimating equations analysis for ICSI fertilization rate and live birth correlated with sperm immobilization method.

	Univariate model		Multivariate model	
	Estimate (95% CI)	*P* value	Estimate (95% CI)	*P* value
ICSI fertilization rate				
Sperm immobilization method (1 vs 0)	0.2884 (0.1484, 0.4283)	**< .0001**	0.2743 (0.1306, 0.4179)	**0.0002**
Female age	0.013 (0.0045, 0.0216)	**0.0029**	0.0102 (− 0.003, 0.0234)	0.1297
Time interval between HCG trigger and ICSI (min)	0.0004 (− 0.0014, 0.0021)	0.6785		
Gravida	− 0.0649 (− 0.1435, 0.0137)	0.1054		
Mature oocyte number	− 0.0026 (− 0.0165, 0.0113)	0.7126		

	Univariate model		Multivariate model	
	Odds ratio (95% CI)	*P* value	Odds ratio (95% CI)	*P* value
Live birth				
Sperm immobilization method (1 vs 0)	9.9832 (1.5997, 62.3024)	**0.0138**	23.4577 (23.396, 23.5215)	**0.0073**
Female age	0.8598 (0.7891, 0.9366)	**0.0005**	0.8212 (0.651, 1.0357)	0.0962
Time interval between HCG trigger and ICSI (min)	0.9969 (0.9835, 1.0104)	0.6474		
Gravida	1.0723 (0.4443, 2.588)	0.8765		
Mature oocyte number	1.4936 (1.2329, 1.8096)	**< .0001**	1.2618 (0.8359, 1.9045)	0.2684

Significant values are in bold.

*Sperm immobilization method 1* aggressive sperm immobilization, *Sperm immobilization method 0* standard sperm immobilization, *HCG* human chorionic gonadotropin, *ICSI* intracytoplasmic sperm injection.

## Discussion

In this study, our data showed that aggressive SI can significantly improve FR, the available number of embryos transferred per ET cycle, and eventually, the LBR in patients with a history of ICSI FF. No significant difference was observed in the number of good-quality embryos per transfer cycle between the standard and aggressive SI groups. The majority of embryos in our study were transferred at the day 3 stage, with an average of 2.6 ± 0.9 in the aggressive SI group and 1.9 ± 0.9 in the standard group. There were twenty-one day 3 embryo transfer (ET) cycles in the aggressive SI group and seventeen day 3 ET cycles in the standard SI group. Only three cases involved the utilization of day 5–6 embryos, with two in the aggressive SI group and one in the standard SI group. Only one blastocyst was transferred in each cycle. The case number of blastocyst transfer is too few to make statistical comparison between two groups.

A significant proportion of our patients had advanced maternal age, with an average age of 38.9 years old in this study. And most of the transferred embryos here were cleavage-stage instead of blastocysts. According to the guidelines of the American Society for Reproductive Medicine^30^, up to 3 cleavage-stage embryos could be considered to transfer. In the standard SI group, the number of embryos available for transfer is lower due to poorer fertilization outcomes. In contrast, aggressive SI significantly improved FR, leading to a greater number of embryos available for transfer per ET cycle. It's worth noting that aggressive SI significantly improved FR, leading to a greater number of embryos available for transfer per ET cycle.

Aggressive SI, performed by compressing the tail of the spermatozoa at the tip up to 15 times, could damage the sperm membrane thoroughly, which may be a mechanism to improve fertilization. Plasma membrane damage of sperms thoroughly before ICSI allow entry and activation of sperm nucleus-decondensing factor which is released from the oocyte^13,14^. Subsequently, sperm-associated oocyte-activating factors can be released into the ooplasm to induce oocyte activation with calcium release^15–17,34^. However, the molecular mechanisms underlying aggressive SI still requires further investigation.

Different immobilization procedures have been used to induce permeabilization of the sperm membrane. The traditional method for immobilization involves compressing the tail of the spermatozoon at the bottom of a dish using an injection pipette in a direction perpendicular to the spermatozoon tail. Compared to the standard method, a higher ICSI FR for immobilized spermatozoa has been noted after more aggressive mechanical procedures in patients with suboptimal semen samples^13,24,35^. Previously, our team revealed similarly higher FR with three different immobilization techniques (dissecting the tail, compressing the mid-piece, and cutting the tail at the mid-portion) compared to the group without SI^24^. Other aggressive techniques include permanently pressing the tail in the mid-piece region, cutting the tail at the mid-portion, and cutting halfway between the head and tip of the spermatozoon^13,14,24^. An RCT conducted by Velaers et al.^14^ compared the standard procedure (pressing the tip of the tail once) and a more aggressive procedure (performing the standard procedure twice along with compression of the sperm mid-piece once) in male infertility patients and found no differences in FR between the two groups. Noteworthily, the rate of good-quality embryo significantly decreased in the aggressive procedure group, which may be related to damage to the mid-piece of spermatozoa, a critical portion that is composed of the centriole and regulates the cleavage patterns of the early embryo^36,37^. Therefore, to avoid damaging the mid-piece of the spermatozoa and affecting the embryo quality, we compressed below the distal half of tail and away from the mid-piece region as a means of SI before ICSI. The quality of the embryos was not impaired in our aggressive treatment group, and there were no documented fetal or congenital anomalies in the offspring.

This study has a few limitations. First, the sample size was relatively small because of the low incidence of ICSI-FF in our patient population. The second limitation was its retrospective nature. Further large-scale RCTs are required to validate the effectiveness of aggressive SI in patients with ICSI-FF. In addition, we did not discuss the male factors of sperm morphology because we lacked related data from the database.

Sperm immobilization including pipetting three to five times onto the upper one-third of the sperm tail have been used for efficient fertilization^18^. However, there remains a lack of guidelines or recommendations regarding the number of tail compressions. Additionally, no difference in fertilization rates (FR) was reported between sperm immobilization methods prior to intracytoplasmic sperm injection (ICSI), comparing triple-touch immobilization to single-touch immobilization^14^. In our clinical practice, we employed a technique where the distal half of the sperm tail was pressed against the dish's lid using a microinjection pipette, applying a maximum of five compressions until immobilization was achieved. Our observations indicated no difference in fertilization rate or clinical pregnancy rate between employing one compression and utilizing five compressions.

There is also currently no established literature regarding the optimal number of compressions required for aggressive sperm immobilization. In the study performed by Palermo, G. D. et al., they didn’t provide absolute number of tail compression but describe the morphology change of sperm in their aggressive immobilization group. In our clinical experience, we utilized a technique where the distal half of the sperm tail was pressed up to 15 times, which also induced similar morphological change with the study by Palermo et al.^13^. The rationale behind selecting 15 compressions in our study is indeed a crucial consideration. Our decision was based on an initial exploration of various compression levels and empirical evidence from our clinical practices. We aimed to strike a balance between achieving effective immobilization and minimizing potential adverse effects. In essence, our study serves as an initial step in exploring the impact of aggressive sperm immobilization, recognizing the need for ongoing research to optimize and standardize this technique with the optimal number of compressions.

However, the strengths of this study were that it was a pioneering and innovative study showing that aggressive SI has a significantly beneficial impact on oocyte fertilization, clinical pregnancy outcomes, and live birth rate in couples with previous ICSI FF. We also applied the GEE model to estimate the parameters of a generalized linear model, with a possible correlation between observations at different time points in the same patient. GEE models account for within-subject correlations and evaluate repeated measurements^31,33^. Moreover, multivariate models were also considered and utilized in this study to adjust for other confounding factors. After adjusting for the effects of age, the time interval between oocyte triggering and ICSI, gravida, and number of retrieved MII oocytes, a significantly higher FR and LBR existed in the aggressive sperm immobilization group.

## Conclusion

For couples with suboptimal semen parameters and unexpected low FR after the traditional ICSI procedure, aggressive SI by pressing the sperm distal tail 15 times prior to ICSI significantly improved the reproductive outcome compared to the standard SI method. Seven uneventful and healthy live babies were delivered, providing convincing evidence for the safety of this technique. Collectively, these data suggest that aggressive SI results in more available embryos for transfer without compromising embryo quality. This technique with compression at the distal half tail of the spermatozoa up to 15 times can be a safe and effective method for patients who experienced poor FR in previous ICSI cycles, without extra cost for pharmacological treatments, such as AOA.

## Data Availability

Data is available upon reasonable request to the corresponding author.
